# 5-Fluoro-2-(2-fluoro­phen­yl)-3-methyl­sulfinyl-1-benzo­furan

**DOI:** 10.1107/S1600536814014810

**Published:** 2014-06-25

**Authors:** Hong Dae Choi, Pil Ja Seo, Uk Lee

**Affiliations:** a Department of Chemistry, Dongeui University, San 24 Kaya-dong, Busanjin-gu, Busan 614-714, Republic of Korea; b Department of Chemistry, Pukyong National University, 599-1 Daeyeon 3-dong, Nam-gu, Busan 608-737, Republic of Korea

**Keywords:** crystal structure

## Abstract

In the title compound, C_15_H_10_F_2_O_2_S, the dihedral angle between the plane of the benzo­furan ring system (r.m.s. deviation = 0.015 Å) and that of the 2-fluoro­phenyl ring is 28.53 (6)°. In the crystal, mol­ecules are linked by C—H⋯O and C—H⋯F hydrogen bonds, and by π–π inter­actions between the furan and benzene rings of neighbouring mol­ecules [centroid–centroid distance = 3.625 (2) Å], forming a three-dimensional network.

## Related literature   

For background information and the crystal structures of related compounds, see: Choi *et al.* (2009*a* ▶,*b* ▶, 2012 ▶).
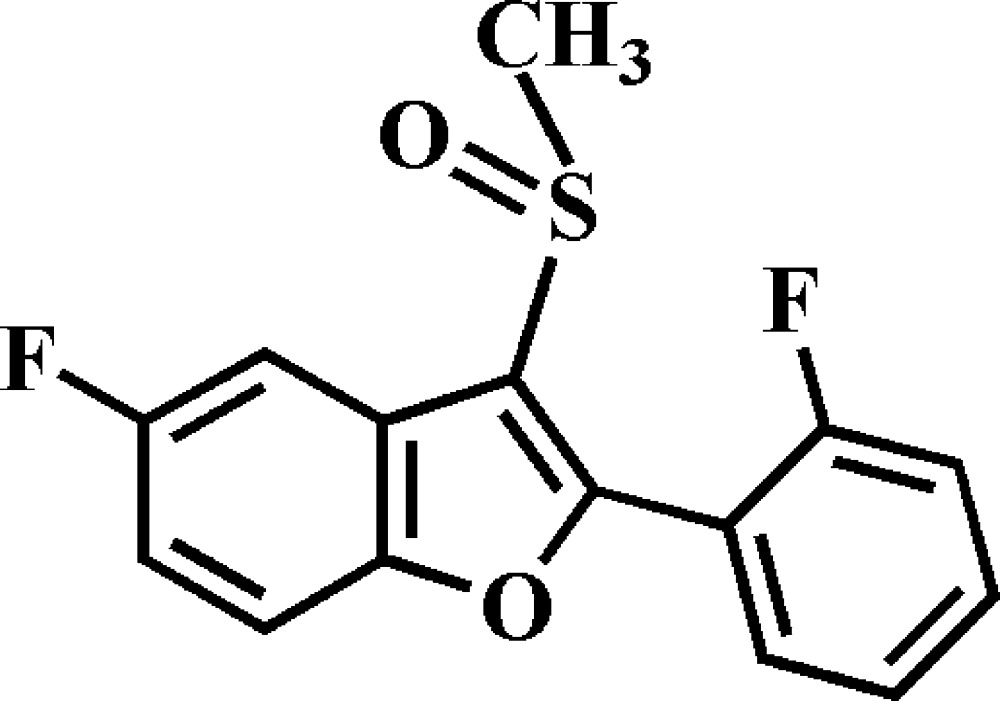


## Experimental   

### 

#### Crystal data  

C_15_H_10_F_2_O_2_S
*M* *_r_* = 292.29
Monoclinic, 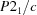

*a* = 8.6184 (2) Å
*b* = 16.8358 (4) Å
*c* = 9.4019 (2) Å
β = 111.254 (1)°
*V* = 1271.40 (5) Å^3^
*Z* = 4
Mo *K*α radiation
μ = 0.28 mm^−1^
*T* = 173 K
0.55 × 0.27 × 0.24 mm

#### Data collection  

Bruker SMART APEXII CCD diffractometer
Absorption correction: multi-scan (*SADABS*; Bruker, 2009 ▶) *T*_min_ = 0.863, *T*_max_ = 0.937
12086 measured reflections
3118 independent reflections
2650 reflections with *I* > 2σ(*I*)
*R*_int_ = 0.029

#### Refinement  

*R*[*F* ^2^ > 2σ(*F* ^2^)] = 0.038
*wR*(*F* ^2^) = 0.100
*S* = 1.07
3118 reflections
182 parameters
H-atom parameters constrained
Δρ_max_ = 0.31 e Å^−3^
Δρ_min_ = −0.40 e Å^−3^

### 

Data collection: *APEX2* (Bruker, 2009 ▶); cell refinement: *SAINT* (Bruker, 2009 ▶); data reduction: *SAINT*; program(s) used to solve structure: *SHELXS97* (Sheldrick, 2008 ▶); program(s) used to refine structure: *SHELXL97* (Sheldrick, 2008 ▶); molecular graphics: *ORTEP-3 for Windows* (Farrugia, 2012 ▶) and *DIAMOND* (Brandenburg, 1998 ▶); software used to prepare material for publication: *SHELXL97*.

## Supplementary Material

Crystal structure: contains datablock(s) I. DOI: 10.1107/S1600536814014810/bt6985sup1.cif

Structure factors: contains datablock(s) I. DOI: 10.1107/S1600536814014810/bt6985Isup2.hkl

Supporting information file. DOI: 10.1107/S1600536814014810/bt6985Isup3.cml

CCDC reference: 1009826

Additional supporting information:  crystallographic information; 3D view; checkCIF report

## Figures and Tables

**Table 1:** Hydrogen-bond geometry (Å, °)

*D*—H⋯*A*	*D*—H	H⋯*A*	*D*⋯*A*	*D*—H⋯*A*
C5—H5⋯O2^i^	0.95	2.52	3.343 (2)	145
C12—H12⋯O2^ii^	0.95	2.39	3.326 (2)	166
C15—H15*A*⋯F1^iii^	0.98	2.54	3.419 (2)	149

Symmetry codes: (i) 
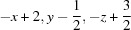
; (ii) 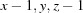; (iii) 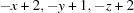.

**Fig. 1.**
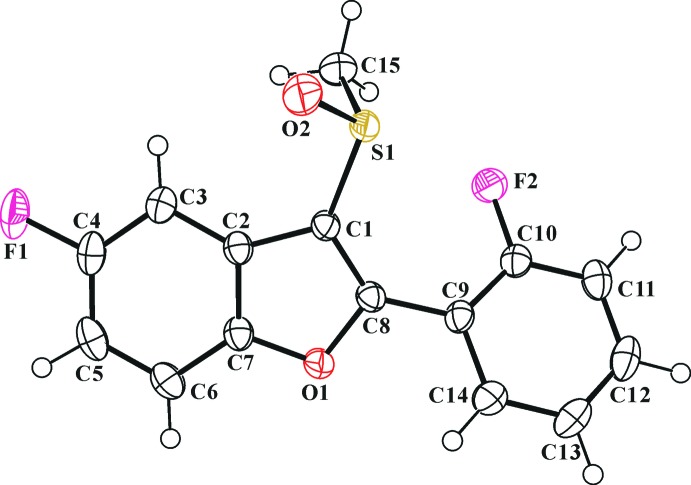
The molecular structure of the title molecule with the atom numbering scheme The displacement ellipsoids are drawn at the 50% probability level. The hydrogen atoms are presented as small spheres of arbitrary radius.

**Fig. 2.**
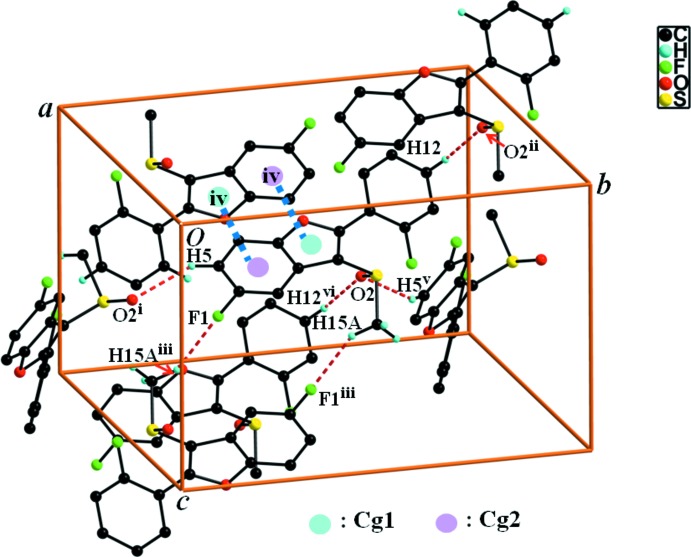
A view of the C—H···O, C—H···F and π–π interactions (dotted lines) in the crystal structure of the title compound. H atoms non-participating in hydrogen-bonding were omitted for clarity. [Symmetry codes: (i) - x + 2, y - 1/2, - z + 3/2; (ii) x - 1, y, z -1; (iii) - x + 2, - y + 1, - z + 2; (iv) - x + 2, - y + 1, - z + 1; (v) - x + 1, y + 1/2, - z + 3/2; (vi) x + 1, y, z + 1.]

## supplementary crystallographic information

### S1. Comment

As a part of our ongoing project of 5-fluoro-3-methylsulfinyl-1-benzofuran derivatives containing 4-bromophenyl (Choi *et al.*, 2009*a*), 4-fluorophenyl (Choi *et al.*, 2009*b*) and 4-methylphenyl (Choi *et al.*, 2012) substituents in 2-position, we report herein on the crystal structure of the title compound.

In the title molecule (Fig. 1), the benzofuran unit is essentially planar, with a mean deviation of 0.015 (1) Å from the least-squares plane defined by the nine constituent atoms. The 2-fluorophenyl ring is essentially planar, with a mean deviation of 0.053 (1) Å from the least-squares plane defined by the six constituent atoms. The dihedral angle formed by the benzofuran ring system and the 2-fluorophenyl ring is 28.53 (6)°. In the crystal structure (Fig. 2), molecules are inked by C—H···O and C—H···F hydrogen bonds (Table 1), and by π–π interactions between the furan and benzene rings of neighbouring molecules, with a Cg1···Cg2^iv^ distance of 3.625 (2) Å and an interplanar distance of 3.295 (2) Å resulting in a slippage of 1.511 (2) Å (Cg1 and Cg2 are the centroids of the C1/C2/C7/O1/C8 furan ring and the C2–C7 benzene ring, respectively), forming a three-dimensional network.

### S2. Experimental

3-Chloroperoxybenzoic acid (77%, 269 mg, 1.2 mmol) was added in small portions to a stirred solution of 5-fluoro-2-(2-fluorophenyl)-3-methylsulfanyl-1-benzofuran (304 mg, 1.1 mmol) in dichloromethane (35 mL) at 273 K. After being stirred at room temperature for 4h, the mixture was washed with saturated sodium bicarbonate solution and the organic layer was separated, dried over magnesium sulfate, filtered and concentrated at reduced pressure. The residue was purified by column chromatography (hexane–ethyl acetate, 1:1 *v*/*v*) to afford the title compound as a colorless solid [yield 73%, m.p. 412–413 K; *R*_f_ = 0.46 (hexane–ethyl acetate, 1:1 *v*/*v*)]. Single crystals suitable for X-ray diffraction were prepared by slow evaporation of a solution of the title compound in acetone at room temperature.

### S3. Refinement

All H atoms were positioned geometrically and refined using a riding model, with C—H = 0.95 Å for aryl and 0.98 Å for methyl H atoms, respectively. *U*_iso_ (H) = 1.2*U*_eq_ (C) for aryl and 1.5*U*_eq_ (C) for methyl H atoms. The positions of methyl hydrogens were optimized using the SHELXL-97 command AFIX 137 (Sheldrick, 2008).

### Crystal data

**Table:**

C_15_H_10_F_2_O_2_S	*F*(000) = 600
*M**_r_* = 292.29	*D*_x_ = 1.527 Mg m^−^^3^
Monoclinic, *P*2_1_/*c*	Melting point = 413–412 K
Hall symbol: -P 2ybc	Mo *K*α radiation, λ = 0.71073 Å
*a* = 8.6184 (2) Å	Cell parameters from 4357 reflections
*b* = 16.8358 (4) Å	θ = 2.4–27.8°
*c* = 9.4019 (2) Å	µ = 0.28 mm^−^^1^
β = 111.254 (1)°	*T* = 173 K
*V* = 1271.40 (5) Å^3^	Block, colourless
*Z* = 4	0.55 × 0.27 × 0.24 mm

### Data collection

**Table:**

Bruker SMART APEXII CCD diffractometer	3118 independent reflections
Radiation source: rotating anode	2650 reflections with *I* > 2σ(*I*)
Graphite multilayer monochromator	*R*_int_ = 0.029
Detector resolution: 10.0 pixels mm^-1^	θ_max_ = 28.3°, θ_min_ = 2.4°
φ and ω scans	*h* = −10→11
Absorption correction: multi-scan (*SADABS*; Bruker, 2009)	*k* = −22→19
*T*_min_ = 0.863, *T*_max_ = 0.937	*l* = −12→12
12086 measured reflections	

### Refinement

**Table:**

Refinement on *F*^2^	Primary atom site location: structure-invariant direct methods
Least-squares matrix: full	Secondary atom site location: difference Fourier map
*R*[*F*^2^ > 2σ(*F*^2^)] = 0.038	Hydrogen site location: difference Fourier map
*wR*(*F*^2^) = 0.100	H-atom parameters constrained
*S* = 1.07	*w* = 1/[σ^2^(*F*_o_^2^) + (0.0492*P*)^2^ + 0.3389*P*] where *P* = (*F*_o_^2^ + 2*F*_c_^2^)/3
3118 reflections	(Δ/σ)_max_ = 0.001
182 parameters	Δρ_max_ = 0.31 e Å^−^^3^
0 restraints	Δρ_min_ = −0.40 e Å^−^^3^

### Special details

**Table:**

Geometry. All esds (except the esd in the dihedral angle between two l.s. planes) are estimated using the full covariance matrix. The cell esds are taken into account individually in the estimation of esds in distances, angles and torsion angles; correlations between esds in cell parameters are only used when they are defined by crystal symmetry. An approximate (isotropic) treatment of cell esds is used for estimating esds involving l.s. planes.
Refinement. Refinement of F^2^ against ALL reflections. The weighted R-factor wR and goodness of fit S are based on F^2^, conventional R-factors R are based on F, with F set to zero for negative F^2^. The threshold expression of F^2^ > 2sigma(F^2^) is used only for calculating R-factors(gt) etc. and is not relevant to the choice of reflections for refinement. R-factors based on F^2^ are statistically about twice as large as those based on F, and R- factors based on ALL data will be even larger.

### Fractional atomic coordinates and isotropic or equivalent isotropic displacement parameters (Å^2^)

**Table:**

	*x*	*y*	*z*	*U*_iso_*/*U*_eq_	
S1	0.71915 (5)	0.69054 (2)	0.61445 (4)	0.02927 (12)	
F1	1.13845 (13)	0.42819 (7)	0.91201 (11)	0.0485 (3)	
F2	0.38183 (11)	0.67630 (6)	0.41827 (10)	0.0374 (2)	
O1	0.67546 (12)	0.49850 (6)	0.36067 (11)	0.0281 (2)	
O2	0.89953 (15)	0.70691 (7)	0.69905 (14)	0.0431 (3)	
C1	0.71116 (17)	0.59444 (8)	0.53646 (16)	0.0234 (3)	
C2	0.82307 (17)	0.53035 (9)	0.60821 (17)	0.0250 (3)	
C3	0.94153 (18)	0.51622 (10)	0.75344 (17)	0.0299 (3)	
H3	0.9632	0.5534	0.8344	0.036*	
C4	1.02416 (19)	0.44539 (10)	0.77150 (18)	0.0336 (4)	
C5	1.0016 (2)	0.38969 (10)	0.6577 (2)	0.0361 (4)	
H5	1.0650	0.3421	0.6785	0.043*	
C6	0.8859 (2)	0.40384 (9)	0.5134 (2)	0.0330 (3)	
H6	0.8681	0.3673	0.4320	0.040*	
C7	0.79767 (17)	0.47404 (9)	0.49421 (17)	0.0267 (3)	
C8	0.62565 (17)	0.57246 (9)	0.38947 (16)	0.0242 (3)	
C9	0.49420 (17)	0.60820 (9)	0.25958 (16)	0.0254 (3)	
C10	0.37363 (18)	0.65773 (9)	0.27581 (17)	0.0271 (3)	
C11	0.24304 (19)	0.68798 (10)	0.15475 (19)	0.0324 (3)	
H11	0.1626	0.7215	0.1713	0.039*	
C12	0.2317 (2)	0.66846 (11)	0.00904 (19)	0.0382 (4)	
H12	0.1426	0.6886	−0.0764	0.046*	
C13	0.3500 (2)	0.61954 (12)	−0.01285 (19)	0.0417 (4)	
H13	0.3418	0.6064	−0.1136	0.050*	
C14	0.4797 (2)	0.58969 (11)	0.10995 (18)	0.0351 (4)	
H14	0.5601	0.5562	0.0930	0.042*	
C15	0.6325 (2)	0.66632 (12)	0.7563 (2)	0.0435 (4)	
H15A	0.6984	0.6239	0.8221	0.065*	
H15B	0.5173	0.6484	0.7060	0.065*	
H15C	0.6347	0.7135	0.8181	0.065*	

### Atomic displacement parameters (Å^2^)

**Table:**

	*U*^11^	*U*^22^	*U*^33^	*U*^12^	*U*^13^	*U*^23^
S1	0.0313 (2)	0.0205 (2)	0.0279 (2)	0.00149 (14)	0.00106 (15)	−0.00264 (14)
F1	0.0421 (6)	0.0544 (7)	0.0399 (6)	0.0162 (5)	0.0039 (5)	0.0204 (5)
F2	0.0311 (5)	0.0469 (6)	0.0322 (5)	0.0074 (4)	0.0092 (4)	−0.0046 (4)
O1	0.0285 (5)	0.0232 (5)	0.0278 (5)	0.0023 (4)	0.0045 (4)	−0.0042 (4)
O2	0.0356 (6)	0.0315 (7)	0.0463 (7)	−0.0063 (5)	−0.0041 (5)	−0.0079 (5)
C1	0.0240 (7)	0.0200 (7)	0.0239 (7)	−0.0001 (5)	0.0060 (5)	0.0002 (5)
C2	0.0239 (7)	0.0225 (7)	0.0278 (7)	0.0003 (5)	0.0085 (6)	0.0026 (6)
C3	0.0289 (7)	0.0308 (8)	0.0271 (8)	0.0014 (6)	0.0068 (6)	0.0047 (6)
C4	0.0273 (7)	0.0373 (9)	0.0332 (8)	0.0043 (6)	0.0073 (6)	0.0148 (7)
C5	0.0333 (8)	0.0262 (8)	0.0509 (10)	0.0080 (6)	0.0177 (7)	0.0123 (7)
C6	0.0354 (8)	0.0223 (8)	0.0426 (9)	0.0026 (6)	0.0155 (7)	0.0013 (7)
C7	0.0256 (7)	0.0234 (7)	0.0293 (8)	0.0004 (6)	0.0079 (6)	0.0026 (6)
C8	0.0233 (7)	0.0207 (7)	0.0269 (7)	−0.0001 (5)	0.0071 (6)	−0.0013 (6)
C9	0.0229 (7)	0.0235 (7)	0.0251 (7)	−0.0021 (5)	0.0029 (5)	−0.0002 (6)
C10	0.0250 (7)	0.0253 (7)	0.0277 (7)	−0.0033 (6)	0.0056 (6)	−0.0001 (6)
C11	0.0247 (7)	0.0280 (8)	0.0398 (9)	0.0000 (6)	0.0059 (6)	0.0058 (7)
C12	0.0305 (8)	0.0389 (10)	0.0333 (9)	−0.0031 (7)	−0.0027 (7)	0.0103 (7)
C13	0.0409 (9)	0.0522 (11)	0.0237 (8)	−0.0017 (8)	0.0020 (7)	0.0002 (7)
C14	0.0332 (8)	0.0390 (10)	0.0286 (8)	0.0031 (7)	0.0059 (6)	−0.0041 (7)
C15	0.0510 (11)	0.0494 (11)	0.0300 (9)	0.0126 (9)	0.0143 (8)	−0.0051 (8)

### Geometric parameters (Å, º)

**Table:**

S1—O2	1.4930 (12)	C6—C7	1.381 (2)
S1—C1	1.7675 (14)	C6—H6	0.9500
S1—C15	1.7952 (18)	C8—C9	1.461 (2)
F1—C4	1.3623 (18)	C9—C10	1.383 (2)
F2—C10	1.3521 (17)	C9—C14	1.402 (2)
O1—C8	1.3757 (17)	C10—C11	1.376 (2)
O1—C7	1.3772 (18)	C11—C12	1.377 (2)
C1—C8	1.3606 (19)	C11—H11	0.9500
C1—C2	1.441 (2)	C12—C13	1.383 (3)
C2—C7	1.388 (2)	C12—H12	0.9500
C2—C3	1.397 (2)	C13—C14	1.378 (2)
C3—C4	1.368 (2)	C13—H13	0.9500
C3—H3	0.9500	C14—H14	0.9500
C4—C5	1.383 (3)	C15—H15A	0.9800
C5—C6	1.382 (2)	C15—H15B	0.9800
C5—H5	0.9500	C15—H15C	0.9800
			
O2—S1—C1	105.45 (7)	C1—C8—C9	135.03 (14)
O2—S1—C15	106.22 (8)	O1—C8—C9	114.27 (12)
C1—S1—C15	97.82 (8)	C10—C9—C14	116.59 (14)
C8—O1—C7	106.33 (11)	C10—C9—C8	122.83 (13)
C8—C1—C2	107.22 (13)	C14—C9—C8	120.49 (14)
C8—C1—S1	126.55 (11)	F2—C10—C11	117.86 (14)
C2—C1—S1	124.91 (11)	F2—C10—C9	118.49 (13)
C7—C2—C3	119.55 (14)	C11—C10—C9	123.63 (14)
C7—C2—C1	104.98 (13)	C10—C11—C12	118.45 (15)
C3—C2—C1	135.45 (14)	C10—C11—H11	120.8
C4—C3—C2	115.65 (15)	C12—C11—H11	120.8
C4—C3—H3	122.2	C11—C12—C13	119.99 (15)
C2—C3—H3	122.2	C11—C12—H12	120.0
F1—C4—C3	117.82 (15)	C13—C12—H12	120.0
F1—C4—C5	117.12 (14)	C14—C13—C12	120.69 (16)
C3—C4—C5	125.06 (15)	C14—C13—H13	119.7
C6—C5—C4	119.46 (15)	C12—C13—H13	119.7
C6—C5—H5	120.3	C13—C14—C9	120.65 (16)
C4—C5—H5	120.3	C13—C14—H14	119.7
C7—C6—C5	116.30 (15)	C9—C14—H14	119.7
C7—C6—H6	121.9	S1—C15—H15A	109.5
C5—C6—H6	121.9	S1—C15—H15B	109.5
O1—C7—C6	125.31 (14)	H15A—C15—H15B	109.5
O1—C7—C2	110.75 (12)	S1—C15—H15C	109.5
C6—C7—C2	123.93 (14)	H15A—C15—H15C	109.5
C1—C8—O1	110.69 (12)	H15B—C15—H15C	109.5
			
O2—S1—C1—C8	−131.06 (13)	C1—C2—C7—C6	177.33 (14)
C15—S1—C1—C8	119.65 (14)	C2—C1—C8—O1	−0.41 (16)
O2—S1—C1—C2	34.10 (14)	S1—C1—C8—O1	166.89 (10)
C15—S1—C1—C2	−75.19 (14)	C2—C1—C8—C9	178.28 (15)
C8—C1—C2—C7	1.30 (15)	S1—C1—C8—C9	−14.4 (2)
S1—C1—C2—C7	−166.26 (11)	C7—O1—C8—C1	−0.67 (15)
C8—C1—C2—C3	179.85 (16)	C7—O1—C8—C9	−179.65 (12)
S1—C1—C2—C3	12.3 (2)	C1—C8—C9—C10	−31.3 (3)
C7—C2—C3—C4	−0.5 (2)	O1—C8—C9—C10	147.36 (14)
C1—C2—C3—C4	−178.85 (16)	C1—C8—C9—C14	152.31 (17)
C2—C3—C4—F1	−178.67 (13)	O1—C8—C9—C14	−29.0 (2)
C2—C3—C4—C5	1.7 (2)	C14—C9—C10—F2	179.21 (13)
F1—C4—C5—C6	179.35 (14)	C8—C9—C10—F2	2.7 (2)
C3—C4—C5—C6	−1.0 (3)	C14—C9—C10—C11	0.7 (2)
C4—C5—C6—C7	−0.9 (2)	C8—C9—C10—C11	−175.83 (14)
C8—O1—C7—C6	−177.54 (14)	F2—C10—C11—C12	−178.95 (14)
C8—O1—C7—C2	1.55 (16)	C9—C10—C11—C12	−0.4 (2)
C5—C6—C7—O1	−178.87 (14)	C10—C11—C12—C13	0.0 (3)
C5—C6—C7—C2	2.2 (2)	C11—C12—C13—C14	0.2 (3)
C3—C2—C7—O1	179.41 (12)	C12—C13—C14—C9	0.1 (3)
C1—C2—C7—O1	−1.76 (16)	C10—C9—C14—C13	−0.5 (2)
C3—C2—C7—C6	−1.5 (2)	C8—C9—C14—C13	176.09 (15)

### Hydrogen-bond geometry (Å, º)

**Table:**

*D*—H···*A*	*D*—H	H···*A*	*D*···*A*	*D*—H···*A*
C5—H5···O2^i^	0.95	2.52	3.343 (2)	145
C12—H12···O2^ii^	0.95	2.39	3.326 (2)	166
C15—H15*A*···F1^iii^	0.98	2.54	3.419 (2)	149

Symmetry codes: (i) −*x*+2, *y*−1/2, −*z*+3/2; (ii) *x*−1, *y*, *z*−1; (iii) −*x*+2, −*y*+1, −*z*+2.

## References

[bb1] Brandenburg, K. (1998). *DIAMOND* Crystal Impact GbR, Bonn, Germany.

[bb2] Bruker (2009). *APEX2*, *SADABS* and *SAINT* Bruker AXS Inc., Madison, Wisconsin, USA.

[bb3] Choi, H. D., Seo, P. J. & Lee, U. (2012). *Acta Cryst.* E**68**, o3338. 10.1107/S1600536812044844 PMC3588940 23476176

[bb4] Choi, H. D., Seo, P. J., Son, B. W. & Lee, U. (2009*a*). *Acta Cryst.* E**65**, o2084. 10.1107/S1600536809030190 PMC2970042 21577502

[bb5] Choi, H. D., Seo, P. J., Son, B. W. & Lee, U. (2009*b*). *Acta Cryst.* E**65**, o2608. 10.1107/S1600536809039312 PMC2971451 21578225

[bb6] Farrugia, L. J. (2012). *J. Appl. Cryst.* **45**, 849–854.

[bb7] Sheldrick, G. M. (2008). *Acta Cryst.* A**64**, 112–122. 10.1107/S0108767307043930 18156677

